# The TRAPs From Microglial Vesicles Protect Against *Listeria* Infection in the CNS

**DOI:** 10.3389/fncel.2019.00199

**Published:** 2019-05-07

**Authors:** Chao Wang, Yang Wang, Xiaochen Shi, Xudong Tang, Wei Cheng, Xueyan Wang, Yanan An, Shulin Li, Hongyue Xu, Yan Li, Wenjing Luan, Xuefei Wang, Zhaobin Chen, Mingyuan Liu, Lu Yu

**Affiliations:** ^1^Key Laboratory for Zoonoses Research, Ministry of Education, Institute of Zoonosis, Department of Infectious Diseases, First Hospital of Jilin University, College of Veterinary Medicine, Jilin University, Changchun, China; ^2^Key Lab for New Drug Research of TCM, Research Institute of Tsinghua University in Shenzhen, Shenzhen, China; ^3^West China School of Public Health, Sichuan University, Chengdu, China; ^4^Shenzhen Center for Disease Control and Prevention, Shenzhen, China; ^5^Jiangsu Co-innovation Center for Prevention and Control of Important Animal Infectious Diseases and Zoonoses, Yangzhou, China

**Keywords:** microglia, Listeria, extracellular traps, reactive oxygen species, vesicle

## Abstract

Previous studies have demonstrated that T cells and microglia could fight against cerebral *Listeria monocytogenes* (Listeria); however, their synergistic anti-Listeria mechanisms remain unknown. Following Listeria infection in a culture system, we found that microglia, but not nerve cells, could release extracellular traps (ETs) which originated from microglial vesicles. Specific inhibitor analysis showed that extracellular DNA (eDNA), matrix metallopeptidases (MMP9 and MMP12), citrullinated histone H3, and peptidyl arginine deiminase 2 were the major components of microglial ETs (MiETs) and were also the components of vesicles. Systematic analysis indicated that Listeria-induced MiETs were cytosolic reactive oxygen species (ROS)- and NADPH oxidase (NOX)-dependent and involved ERK. MiETs were exhibited in Listeria-infected mouse brain and might protected against Listeria infection via bacterial killing in a mouse meningitis model, and MiETs existed in cerebrospinal fluid (CSF) from Listeria meningitis patients *in vivo* and *in vitro. A*dditionally, interferon-γ could induce MiET formation in Listeria-infected microglia *in vitro* that was mediated by NOX, and there was a positive relationship between the elevated level of IFN-γ and eDNA and nucleosomes in the brain homogenates and CSF of Listeria meningitis model mice and in the CSF before treatment in clinical Listeria meningitis patients. Together, this is the first report of MiET formation, these findings pave the way for deeper exploration of the innate immune response to pathogens in CNS.

## Introduction

*Listeria monocytogenes* (Listeria) is an intracellular pathogen that causes severe central nervous system (CNS) infection in humans and animals, and Listeria was detected predominantly within macrophages constituting the microglia (de Noordhout et al., 2014). Listeria is well known to move from cell to cell without exposure to the extracellular fluid (Dramsi et al., 1998), thus bypassing the humoral immune system of the organism. Final control is not achieved until the adaptive immune system provides cytotoxic CD8^+^ T cells to lyse cells infected with Listeria (Schluter et al., 1999).

Microglia are a resident mononuclear phagocyte population in the CNS and are gatekeepers of CNS immunology (Kettenmann et al., 2011). Microglia contribute to both local innate and adaptive immune responses, as well as the defense against many pathogens, including bacteria in the brain. In addition, previous studies have revealed that CD8^+^ T cells are responsible for the most important function to remove intracellular parasitic infections in the immune system; thus, T cells and microglia are significant to maintain health in the CNS (Ziv et al., 2006). Notably, like other antigen-presenting cells, microglia also express MHC-I; thus, microglia can be recognized and broken by CD8^+^ T cells (Sedgwick et al., 1991). Previous studies have demonstrated that T cells and microglia could fight against cerebral Listeria (Virna et al., 2006). However, the mechanism underlying how CD8^+^ T cells and microglia coordinate to fight against neurolisteriosis remains elusive.

The formation of extracellular traps (ETs) as a novel antimicrobial mechanism was recently recognized in neutrophils (Brinkmann et al., 2004), macrophages (Aulik et al., 2012), mast cells, eosinophils, and basophils. ET formation is a cell death program identified as ETosis, which is different from other types of cell death (Brinkmann et al., 2004). ETs have been reported *in vivo* and *in vitro* in response to infection with many different pathogens or treatment by some agents, such as phorbol 12-myristate 13-acetate (PMA) (Pilsczek et al., 2010), ionomycin and interferon. The phosphorylation of ERK was the most important pathway involved in NET formation. Notably, ETs have been demonstrated to be double-edged swords for innate immunity. NETs participate in the pathogenesis of inflammatory and autoimmune disorders and are involved in vascular disorders, glomerulonephritis, chronic lung disease, sepsis, and thrombus formation in deep vein thrombosis. However, the significance of ETs makes them worthy of deeper study regardless of their pros and cons.

Vesicles of varying cellular origins have been increasingly recognized for their participation in a range of physiological and pathological processes. Polarized macrophages, including microglia, release vesicles in the extracellular space (Bianco et al., 2005). These vesicles have been used as powerful biomarkers in several pathological processes (Garzetti et al., 2014), such as the promotion of the inflammatory response by vesicles released from macrophages stimulated by bacteria and microglia activation associated with neuroinflammation that causes increased cerebrospinal fluid (CSF) vesicles. Particularly, a recent study reported that neutrophil vesicles could release their content into the extracellular space to form NETs upon *Staphylococcus aureus* stimulation (Pilsczek et al., 2010).

In this study, we corroborated that Listeria could stimulate microglia cells to form vehicles, which further released ETs, and microglial ETs (MiETs) could arrest or kill Listeria *in vitro* and *in vivo*. We analyzed the major components of MiETs compared with those from NETs, the mechanism of the MiET formation, the effect of IFN-γ on MiET formation, and the level of MiET-related eDNA and nucleosomes *in vitro* and in an *in vivo* Listeria mouse model, as well as in clinical bacterial meningitis (BM) patients.

## Materials and Methods

### Bacterial Strains, Cells and Reagent

The following bacteria were used in this study: *Listeria monocytogenes* (ATCC 19111). Listeria (10^8^) treated with FITC (5 μM) for 15 min in PBS. Murine primary microglia, astrocyte, oligodendrocyte and neuron were isolated from neonatal C57BL/6 mouse brains following a routine method (McCarthy and de Vellis, 1980; Hatten, 1985; Sedgwick et al., 1991). The murine microglia cell line (BV2) and human microglia cell line (HMO6) were obtained from American Type Culture Collection (ATCC). CD8^+^ T cells isolated by Miltenyi CD8a (Ly-2) MicroBeads. All the reagent information was shown in Supplementary Table S1.

### Mouse Meningitis Model

Male 10- to 12-week-old C57BL/6 male mice were used for the foundation of the meningitis model. Meningitis was induced by transcutaneous intracisternal injection of 10 μl of a bacterial suspension containing 10^5^ colony-forming units (CFU) of Listeria or PBS. Mice were monitored every 4–6 h (starting from 12 h after inoculation), and the clinical manifestations were observed including activity, respiration rate, breathing, food intake and neurological signs (Mook-Kanamori et al., 2012; Koopmans et al., 2018). Any animal that appeared moribund prior to the designated endpoint would be humanely euthanized, and death was recorded as occurring the next day. After 24 h, CSF was collected by puncture of the cisterna magna. The brain was removed after euthanasia. Half of each brain was used for paraffin sections for immunohistochemical analysis. The other homogenized brain half and the CSF were serially diluted 10-fold and plated on LB agar plates.

The mice were kept to a controlled 12-h light/dark cycle, and food and water were provided *ad libitum* according to our institutional guidelines. The experiments were approved by the Jilin University Animal Care and Use Committee (No.: IZ-2009-008).

### Patients and Control Subjects

Cerebrospinal fluid samples (*n* = 9) were collected from patients reporting to the First Hospital of Jilin University and Changchun Infectious Disease Hospital. BM was diagnosed through bacterial culture, bacterial smears. Nine patients were diagnosed with Listeria meningitis; additionally, six control cases were included, who had been suspected of CNS infection but were excluded meningitis in the end. The patients hospitalized in the institution and suspected of having infection in CNS. They provided written consent prior to lumbar puncture. All the clinical information of the nine patients are presented in Supplementary Table S2. The procedures were in agreement with the regulations and ethical standards of the two hospitals for the collection of specimens and basic clinical information, written informed consent was given by all patients who participated in this study.

### CD8+ T Cell-Mediated Cytotoxicity Experiment

The CD8^+^ T cells (6 × 10^5^ cells) were incubated with Listeria*-*infected (FITC, MOI = 50:1) BV2 cells (6 × 10^5^ cells) in confocal dish with serum-free and antibiotic-free medium for 2 h (Karttunen et al., 1992). The results were examined using laser-scanning confocal microscope (CLSM, Olympus FluoView FV1000).

### Confocal Microscopy and Immunofluorescence Staining

Microglia were seeded onto 24-well glass chamber slides (2 × 10^5^ cells/well) and stimulated with the indicated reagents or infected with bacteria for 2 h. Immunofluorescence staining was performed using the routine method (Papayannopoulos et al., 2010). The results were examined using a fluorescence microscope (Olympus BX53) and a laser-scanning confocal microscope (CLSM, Olympus FluoView FV1000). Image analyses and export were performed using a Fluoview ver. 1.7.3.0 (Olympus, Japan).

### Scanning Electron Microscopy (SEM)

BV2 cells were plated onto glass cover slides and infected with Listeria (MOI = 50:1) as described for the fluorescence microscopy experiments (Aulik et al., 2012). After 2 h of incubation, the samples were prepared for SEM. Finally, the samples were coated with gold using a sputter coater and examined using a JEOL JSM-7000F (Jeol, Japan).

### Transmission Electron Microscopy (TEM)

To perform TEM, BV2 cells were washed twice with PBS and collected at the bottom of 1.5 ml Eppendorf tubes following centrifugation at 1500 rpm for 5 min; then, the samples were treated for TEM (Garzetti et al., 2014). The cells and ETs were examined under a transmission electron microscope (Hitachi H-7650, Japan).

### Flow Cytometry

BV2 cells were characterized by staining with the following antibodies: rabbit anti-mouse CD86-PE and rabbit anti-mouse CD206-FITC. CD8^+^ T cells stained by mouse anti-Human CD8a-Cy7. The samples were detected with FACS caliber flow cytometer (BD Biosciences, CA). The data acquired were analyzed with FlowJo (Treestar software, United States).

### Vesicle Collection

The supernatant of BV2 cells induced by Listeria was centrifuged at 2,000 × *g* for 10 min, 4,000 × *g* for 15 min and 15,000 × *g* for 30 min at 4°C to remove cellular debris. Each supernatant was then ultra-centrifuged at 100,000 × *g* for 90 min at 4°C, and the pellet was collected. The vesicle fractions were resuspended in PBS and were stored at –80°C until use (Palazzolo et al., 2012).

### Western Blot Analysis

For the detection of protein expression levels, western blotting was performed according to the routine method (Papayannopoulos et al., 2010). The images were obtained using a CanoScan LiDE 100 scanner (Canon). The results were quantified with Image-J software.

### Bactericidal Activity Assays

Listeria was added to BV2 cells and human microglia at an MOI of 50:1 after treatment with 10 μg/ml cytochalasin D for 20 min. Control wells were prepared with bacteria but without cells. The assay was performed as previously described (Grinberg et al., 2008). The percentage of killing by MiETs in replicate wells containing cytochalasin D to inhibit phagocytosis was determined using the equation: [1 – (CFU_extracellular_/CFU_control_)] × 100%.

### Determination of eDNA and Nucleosome Levels and IFN-γ Production

eDNA levels were determined in the cell samples, mouse brain homogenates, mouse CSF supernatants and patient CSF supernatants. ODs were measured with a spectrofluorophotometer (Infinite F200 Pro, Switzerland) using 480-nm excitation and 545-nm emission wavelengths. Background fluorescence of PBS (with SytoxGreen) was subtracted from all samples.

The levels of IFN-γ and nucleosomes from mouse brain homogenates, mouse CSF supernatants and patient CSF supernatants were quantified using the Mouse Interferon-γ ELISA Kit (Cusabio, United States), Human Interferon-γ ELISA kit (Life Technologies, United States), and Cell Death Detection ELISAPLUS kit (Roche) in accordance with the manufacturers’ suggestions. The nucleosome assay allowed the relative quantification of histone-complexed DNA fragments (mono- and oligo-nucleosomes).

### Detection of Cytosolic Reactive Oxygen Species (ROS) and Mitochondrial ROS

For cytosolic ROS detection, we used dihydrorhodamine (DHR) 123 (a fluorescent indicator of cytosolic ROS). Cells were stimulated with the indicated reagents or infected with bacteria and then incubated with DHR 123 for 20 min without light. For mitochondrial ROS detection, a MitoSOX Red superoxide indicator (Molecular Probes, United States) was used. The results were obtained with a spectrofluorophotometer.

### Detection of MiETs Produced *in vivo* and *in vitro*

To quantify MiETs produced *in vivo*, CSF samples from mice and patients were incubated in 24-well glass chamber slides for 4 h in the presence of 100 μM of Cl-amidine to prevent continued MiET production. For *in vitro* analysis, the same volume of CSF samples was incubated in the absence of Cl-amidine to allow the continued generation of MiETs. Marking MiETs by staining DNA (the main component in MiETs), and the DNA was specifically stained by using Hoechst 33342 and SYTOX Orange. Microglia were labeled with the surface marker protein Iba1 of microglia. MiETs and microglia were co-localized. The plates were used for immunofluorescence staining or plate reader assays as described above.

### Statistical Analysis

All statistical analyses were performed using GraphPad Prism (Version 5.01, GraphPad Prism software, Inc., San Diego, CA, United States). The data are representative of triplicate experiments and presented as the mean value ± the SD. Significance was assessed using Student’s *t*-test. Multiple intervention experiments are compared with one-way ANOVA followed by Tukey’s post-test correction. For *p* values, ^∗^*p* < 0.05, ^∗∗^*p* < 0.01, or ^∗∗∗^*p* < 0.001 compared with the control were considered statistically significant.

## Results

### Listeria-Induced MiET Production

**FIGURE 1:**
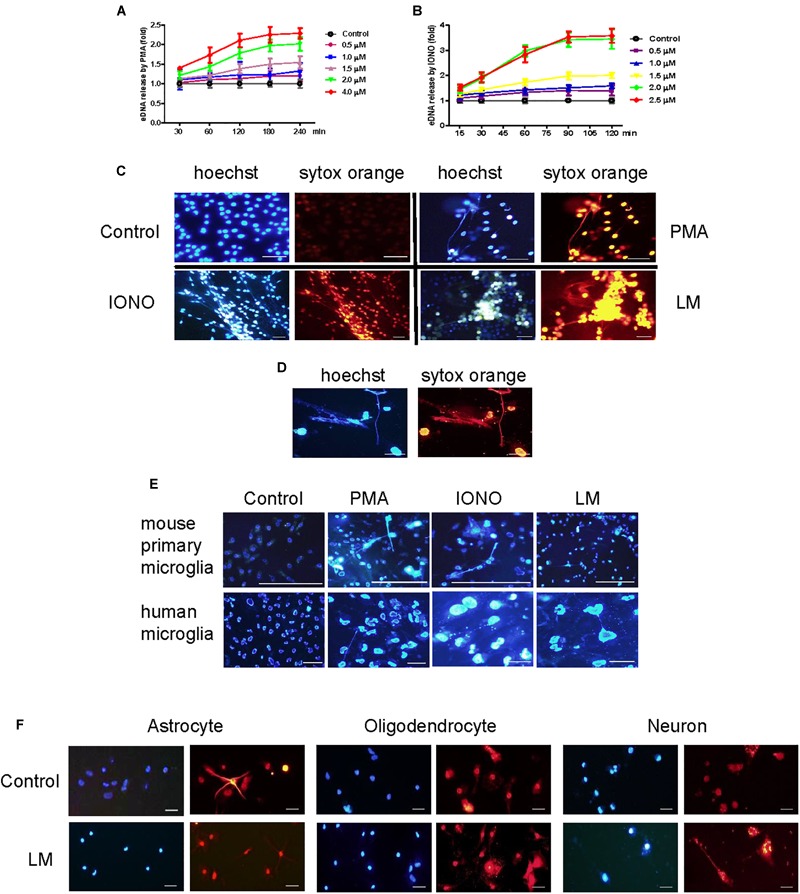
Listeria induced MiETs production. A series of PMA concentrations (0.5–4 μM) were used to treat the BV2 cells for different times (30–240 min). The cells were stained with 1 μM Sytox green for 10 min and then detected with a plate reader **(A)**. Ionomycin at different doses (0.5–2.5 μM) was used to treat BV2 cells for 15–120 min **(B)**. PMA (2 μM), ionomycin (1.5 μM) and Listeria (MOI = 50:1) were added to BV2 cells and incubated in 24-well glass bottom plates (2 × 10^5^ cells/well). The cells were stained with Hoechst 33342 and Sytox orange and then observed by fluorescence microscopy **(C)**. The CD8^+^ T cells (6 × 10^5^ cells) were incubated with Listeria-infected (MOI = 50:1) BV2 cells (6 × 10^5^ cells) in confocal dish. The cells were stained with Hoechst 33342 and Sytox orange and then observed by fluorescence microscopy **(D)**. Listeria (MOI = 50:1), PMA (2 μM) and ionomycin (1.5 μM) were added to mouse primary microglia and a human microglia cell line and the results were collected by fluorescence microscopy **(E)**. Listeria (MOI = 50:1) was added to mouse primary astrocytes, oligodendrocyte and neuron and the results were collected by fluorescence microscopy **(F)**. Scale bars = 50 μm.

To investigate whether microglia could release ETs, two positive potent ET inducers (PMA and ionomycin) were used to treat BV2 cells. PMA induced eDNA release (a main component of ET production) in a dose- and time-dependent manner in BV2 cells within 4 h of stimulation (Figure 1A,B). However, ionomycin caused a more rapid dose- and time-dependent increase in DNA fluorescence within 90 min of treatment (Figure 1B,C). Next, Listeria was used to infect BV2 cells and significantly induced eDNA release at the respective MOIs at 3 h. These results imply that Listeria contributed to MiET production. Moreover, we added Listeria released from microglia broken by CD8^+^ T cells to new microglia; it was found that this released Listeria also induced BV2 cells to produce MiETs (Figure 1D). Next, we found that Listeria, PMA and ionomycin could induce mouse primary microglia and HMO6 cells to release eDNA (Figure 1E and Supplementary Figures S5A,B). Meanwhile, it was also found that astrocytes, oligodendrocytes and neurons did not release eDNA under Listeria infection (Figure 1F), indicating that ET formation was the special nature of microglia in the brain, rather than that in other nerve cells.

### Listeria Transmits Between Microglia and CD8+ T Cells Break Infected Microglia

**FIGURE 2:**
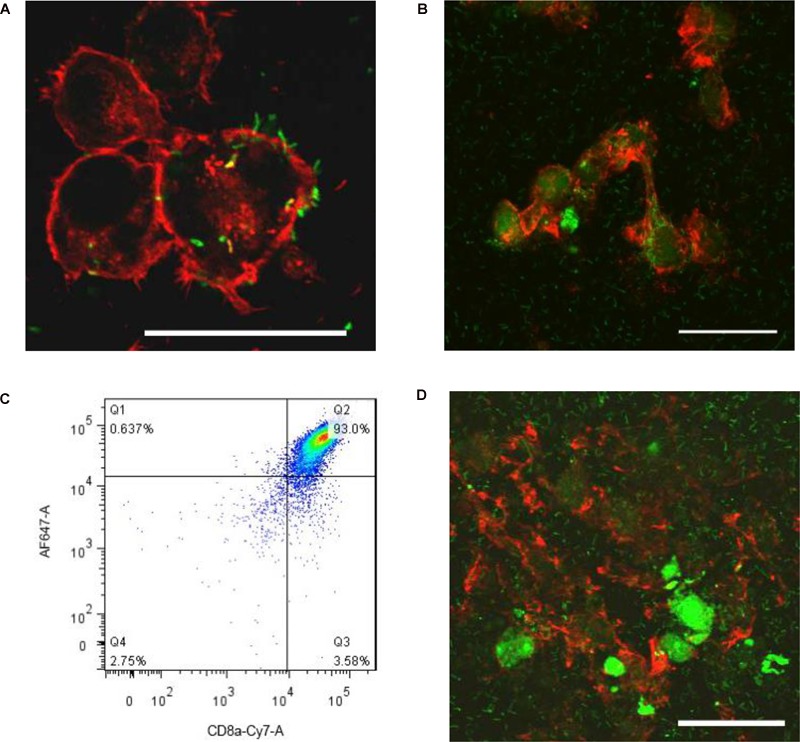
Listeria is transmitted between microglia. BV2 cells were incubated in a confocal dish with serum-free and antibiotic-free medium. Listeria (FITC, MOI = 50:1) was added to BV2 cells and then was incubated in a confocal dish (6 × 10^5^ cells). By confocal microscopy, Listeria (arrow) was observed to penetrate the BV2 cells (Rhodamine phalloidin) **(A)**. Listeria (arrow) spread from cell to cell by remaining intracellular **(B)**. CD8^+^ T cells were inspected by flow cytometry **(C)**. The extracted CD8^+^ T cells (6 × 10^5^ cells) were added to the confocal dish; after 2 h, most of the cells were broken, and Listeria entered the culture medium **(D)**. Scale bars = 50 μm.

Listeria is well known to move from macrophages to ambient macrophages without exposure to the extracellular fluid (Dramsi et al., 1998). To verify whether Listeria can also spread between microglia, we established a Listeria infection model *in vitro*, and then FITC-labeled Listeria was incubated with BV2 cells. Listeria was located in the cytoplasm of most infected microglia (Figure 2A); some of the bacteria also existed in the cell extension part of two adjacent cells (Figure 2B), indicating that Listeria could spread from cell to cell in the microglia without exposure to the extracellular fluid. Furthermore, to confirm whether CD8^+^ T cells can recognize and break Listeria-infected microglia, CD8^+^ T cell-mediated cytotoxicity was performed, and it was found that the purified CD8^+^ T cells from mice broke most of the Listeria-infected BV2 cells and released the Listeria into the culture medium (Figure 2C,D). Together, these results suggest that Listeria could transmit between microglia, and the Listeria-infected microglia could be destroyed by CD8^+^ T cells, followed by release of the bacteria into the extracellular matrix (Supplementary Figure S5C).

### Identification of MiET Structures and MiET Originated From Vesicles

**FIGURE 3:**
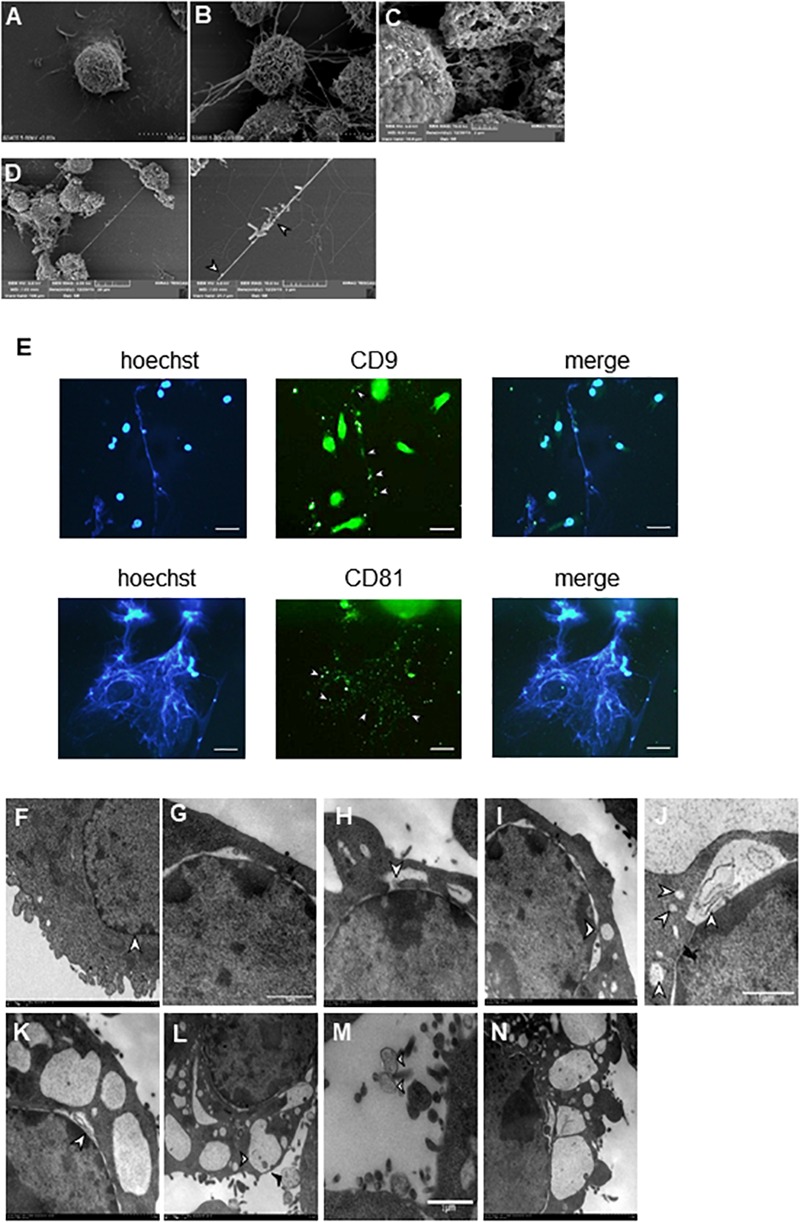
Identification of MiET structures originated from vesicles. BV2 cells were seeded onto poly-lysine-coated glass slides for scanning electron microscopy. Cells without treatment were used as the control **(A)**. Other cells were treated with 2 μM PMA **(B)** for 3 h, ionomycin **(C)** for 2 h or Listeria (MOI = 50:1) **(D)** for 3 h. Listeria (**D**, arrow) was strung in a line or entrapped by the net structures, and some vesicle-like structures were around the net structures (**B–D**, arrowhead). BV2 cells were infected with Listeria (MOI = 50:1) for 3 h. The results were examined using a fluorescence microscope showing MiETs (blue) and vesicles (green) **(E)**. Scale bars = 50 μm. BV2 cells were infected with Listeria (MOI = 50:1) or treated with PMA (2 μM) for 3 h. Transmission electron micrographs showing the complete cell nucleus without treatment were used as the negative control **(F)**. Listeria-infected cells are shown with separated inner and outer nuclear membranes **(G)**. The DNA strands could be seen in the gap between the nuclear membranes **(H)**. NPCs could also be seen **(I)**. A DNA-containing vesicle was seen budding off the nuclear envelope **(J)**. The nuclei were surrounded by vesicles containing DNA strands, and the plasma membranes were intact **(K)**. Vesicles were seen fusing with the outer plasma membrane (**L**, arrowhead), and some were released directly into the extracellular space (**L**, arrow). Vesicles (**M**, arrowhead) were lysed and emptied their contents to form MiETs (**M**, arrow) in response to Listeria. PMA-treated cells showed similar changes as Listeria-infected cells (**N**).

Our SEM results showed that BV2 cells stimulated with Listeria, PMA, or ionomycin released extensive extracellular networks and some vesicle-like structures (Figure 3A–D). Many bacteria were strung in a line or entrapped by the net structures induced by the bacteria (Figure 3D). Immunofluorescence staining results showed that some vesicles (CD9^+^ and CD81^+^) hung among the fibers of Listeria-induced MiETs in 3 h (Figure 3D,E), we speculated that MiETs may be released by vesicles. Next, we used TEM to further investigate the morphological changes in the nuclear structure related to Listeria-induced MiET formation in 3 h. It was revealed that the inner and outer nuclear membranes of the microglia separated under the condition of Listeria infection (Figure 3G), and DNA strands could be seen in the gaps between the nuclear membranes (Figure 3H); by contrast, no gap between the inner and outer nuclear membranes was observed in the uninfected microglia (Figure 3F). The nuclear pore complex (NPC) has been regarded as a marker of the inner and outer nuclear membranes (Garzetti et al., 2014). After stimulation with Listeria, intact NPCs were observed during vesicle formation accompanied by the separation of the inner and outer nuclear membranes of microglia (Figure 3I). Additionally, many DNA-containing vesicles were seen budding off the nuclear envelope (Figure 3J). However, in cells with dilated nuclear envelopes, the nuclei were surrounded by vesicles containing DNA strands despite the presence of intact plasma membranes (Figure 3K). Additional vesicles were released from the nuclear envelope and were fused with the plasma membrane, whereas some of the vesicles were released directly into the extracellular space (Figure 3L). Moreover, some of the vehicles were lysed and emptied their contents to form MiETs outside the cell in response to Listeria (Figure 3M). Three hours of PMA stimulation resulted in morphological changes in the nuclear structure similar to those induced by Listeria (Figure 3N). Together, these results suggest that Listeria-induced MiET formation originated from vesicles.

### eDNA, Matrix Metallopeptidases (MMP9 and MMP12), Citrullinated Histone H3 and PAD2 Are Major Components of MiETs

**FIGURE 4:**
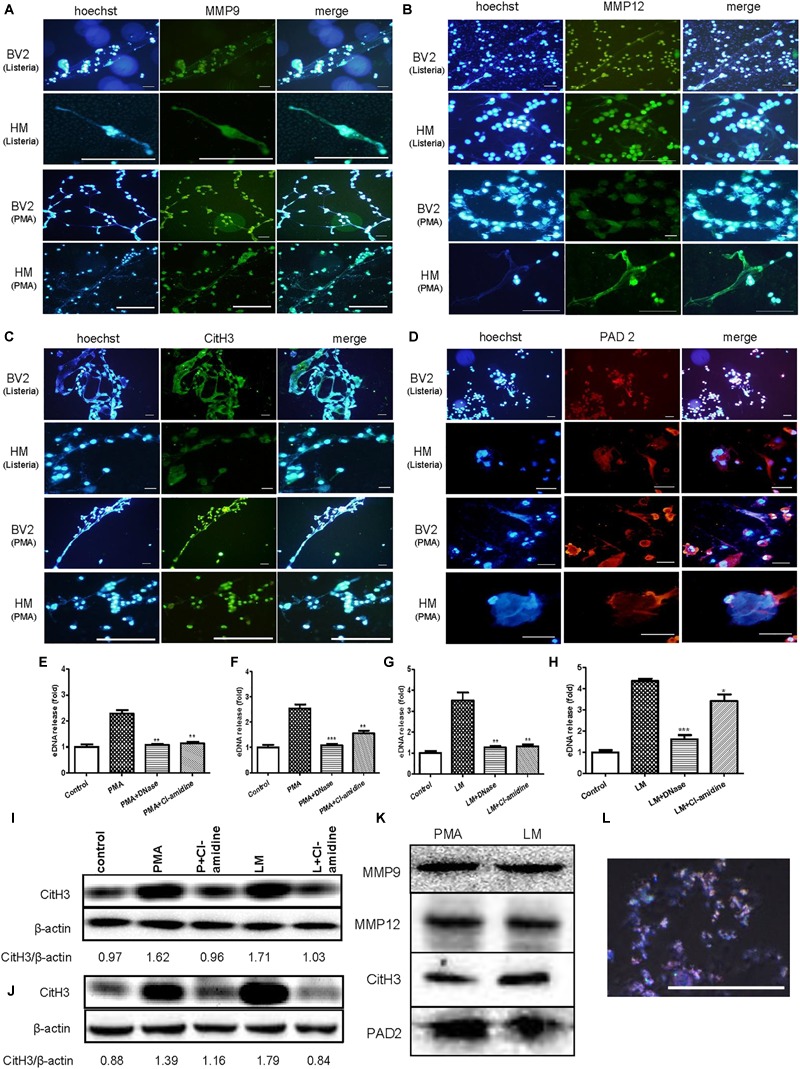
eDNA, MMPs, Citrullinated histone H3 and PAD2 are major components of MiETs. Anti-MMP9 antibody **(A)**, anti-MMP12 antibody **(B)** and eDNA staining of mouse and human microglia following PMA treatment or Listeria treatment. Anti-histone H3 antibody and eDNA staining of the two cell lines following PMA treatment or Listeria treatment **(C)**. Anti-PAD2 antibody and eDNA staining of the two cell lines following PMA treatment or Listeria treatment **(D)**. Scale bars = 50 μm. Mouse or human microglia were pre-treated with DNase I (40 U/ml) or Cl-amidine (100 μM). Next, mouse microglia were exposed to PMA **(E)** or Listeria **(G)**. Pre-treated human microglia were also incubated with PMA **(F)** or Listeria **(H)**. The cells were stained with Sytox green and were detected by a plate reader. ^∗^*p* < 0.05, ^∗∗^*p* < 0.01, ^∗∗∗^*p* < 0.001, compared with the PMA- or Listeria-treated cells. The data are representative of triplicate experiments and are presented as the mean value ± SEM. Mouse microglia **(I)** and human microglia **(J)** were pre-treated with 100 μM Cl-amidine for 30 min. Next, PMA or Listeria was added to the cells for 2 h. Western blotting was used to assess the citrullination of histone H3. The western blotting results demonstrated that MMP9, MMP12, citrullinated histone H3 and PAD2 were the components in the vesicles **(K)**. Vesicles marked by rabbit anti-mouse CD9-AF647 antibody (red) and DNA stained with Hoechst 33342 (blue), as observed by confocal microscopy **(L)**.

To explore the major components of MiETs, the existence of eDNA, MMP9, MMP12, citrullinated histone H3 and PAD2 in MiETs was detected. The release of eDNA webs was observed in Listeria-infected or PMA-treated mouse and human microglia (Figure 4A–D). eDNA colocalized with MMP9 or MMP12 (Figure 4A,B), citrullinated histone H3 (Figure 4C), and PAD2 (Figure 4D) in mouse and human MiETs. DNase I treatment abolished Listeria- or PMA-induced MiET formation (Figure 4E–H), confirming that MiETs contained eDNA.

Moreover, our results showed that pre-treatment with the PAD inhibitor Cl-amidine inhibited the formation of Listeria- or PMA-induced MiETs in mouse and human microglia (Figure 4E–H). Moreover, western blotting demonstrated that Cl-amidine treatment inhibited histone H3 citrullination in bacteria-infected or PMA-treated mouse and human microglia (Figure 4I,J). These data indicated that PAD activity is required for histone citrullination and MiET formation. Together, these results confirmed that microglia could release MiETs and that eDNA, MMP9, MMP12, citrullinated histone H3 and PAD2 were the main MiET components.

Furthermore, the western blotting results showed that the vesicles from the cultures of Listeria-infected microglia contained MMP9, MMP12, citrullinated histone H3 and PAD2 (Figure 4K), and Hoechst 33342 and CD9 fluorescent antibody staining showed that the vesicles contained DNA (Figure 4L), all suggesting that the vesicles contained components similar to those of MiETs.

### Microglia Tend to M1-Like Polarity While MiETs Were Induced by Listeria

To investigate the polarization of microglia, when Listeria induced microglia to produce MiETs, we detected the phenotypes in BV2 cells with or without Listeria infection. The results showed that Listeria infection increased the expression of CD86 (M1 marker) in microglia. However, CD206 (M2 marker) expression was inhibited by Listeria infection on BV2 cells (Supplementary Figure S1A). The western blotting results verified this polarization tendency; CCR7 expression was significantly higher after Listeria infection than in the control group, while the results of the expression of the M2 marker Arg-1 was opposite (Supplementary Figure S1B). Additionally, the ELISA results showed that the level of the M1 marker TNF-α and IL-1β was significantly higher in the supernatant of Listeria-infected BV2 cells than that in the control group (*p* < 0.001), while there was no difference in the level of the M2 marker IL-10 between the Listeria-infected group and control group (Supplementary Figure S1C). Taken together, these results indicate that microglia tend to M1-like polarity when MiETs were induced by Listeria (Supplementary Figure S5D).

### Listeria Killing by MiETs *in vitro*

**FIGURE 5:**
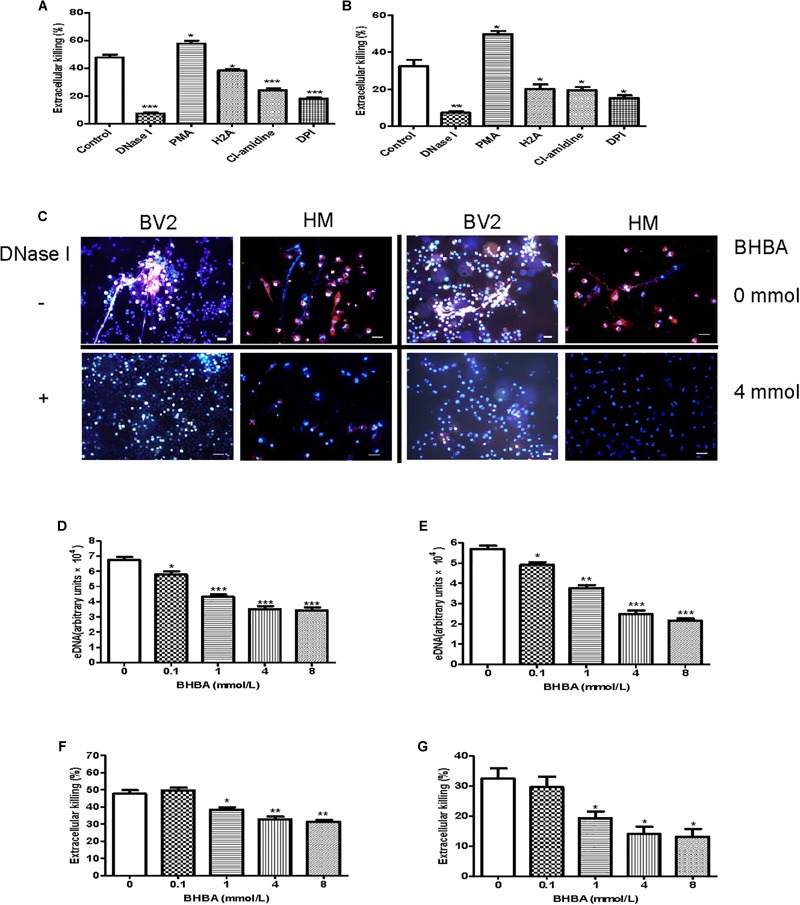
Listeria killing by MiETs *in vitro*. BV2 cells and human microglia were seeded into 24-well flat bottom plates (2 × 10^5^) and then pre-treated with DNase I (40 U/ml), H2A (1 μg/ml) or Cl-amidine (100 μM) for 1 h. The cells were infected with Listeria (MOI = 50:1) or Listeria and PMA (2 μM) for 2 h after treatment with 10 μg/ml cytochalasin D for 20 min. Supernatants and cell lysates of BV2 cells **(A)** or human microglia **(B)** were collected for CFU counting and the calculation of the extracellular killing rate. Pictures showed Listeria-infected BV2 cells and human microglia with or without DNase I (40 U/ml) or BHBA treatment (4 mM) **(C)**. The two cell lines were pre-treated with a series of concentrations of BHBA ranging from 0.1 to 8 mM for 20 min and then infected with Listeria. A plate reader was used to quantitate eDNA contents in BV2 cells **(D)** and human microglia **(E)**. The extracellular killing rate was calculated similar to the method described for BV2 cells **(F)** and human microglia **(G)**. ^∗^*p* < 0.05, ^∗∗^*p* < 0.01, ^∗∗∗^*p* < 0.001, compared with the control cells. The data are representative of triplicate experiments and presented as the mean value ± the SEM. eDNA was stained with Hoechst 33342 and Sytox orange. Scale bars = 50 μm.

Additionally, we investigated whether the Listeria entrapped in MiETs were killed *in vitro*. To differentiate between bactericidal and phagocytic activity of ETs, we interdicted phagocytosis using the actin-polymerization inhibitor CytD prior to bacterial infection. The phagocytosis inhibition assay showed that mouse and human microglia could kill approximately 48 and 32% of Listeria, respectively (Figure 5A,B). The addition of PMA or DNase significantly increased or decreased the amount of extracellular Listeria, respectively (Figure 5A–C). These results implied that MiETs might kill extracellular Listeria *in vitro*, whereas the split of eDNA emancipated the bacteria entrapped within the DNA fibrils and abrogated the MiET-mediated killing. Pre-treatment with anti-histone antibody (H2A), PADs inhibitor Cl-amidine, or NADPH oxidase (NOX) inhibitor DPI significantly inhibited the extracellular killing of Listeria by microglia (Figure 5A,B). These data suggested that histone, hypercitrullination of histone H3 mediated by PAD 2, and NOX were necessary for the killing of Listeria. Moreover, the ketone body β-hydroxybutyrate (BHBA) was also found to inhibit MiET formation in a dose-dependent manner and significantly decreased the killing of extracellular Listeria by mouse and human microglia (Figure 5D–G). Ketones are known to constitute an important fraction of fuel for consumption by the brain. Recent report demonstrated that the concentration of BHBA in children on the ketogenic diet might reach up to 1 mM (Grinberg et al., 2008). Our result suggested that high BHBA levels in the brains of ketogenic fasting children might affect MiET formation and lead to a decrease in the bactericidal effect of microglia in the CNS.

### NOX-Dependent and NOX-Independent MiETs

**FIGURE 6:**
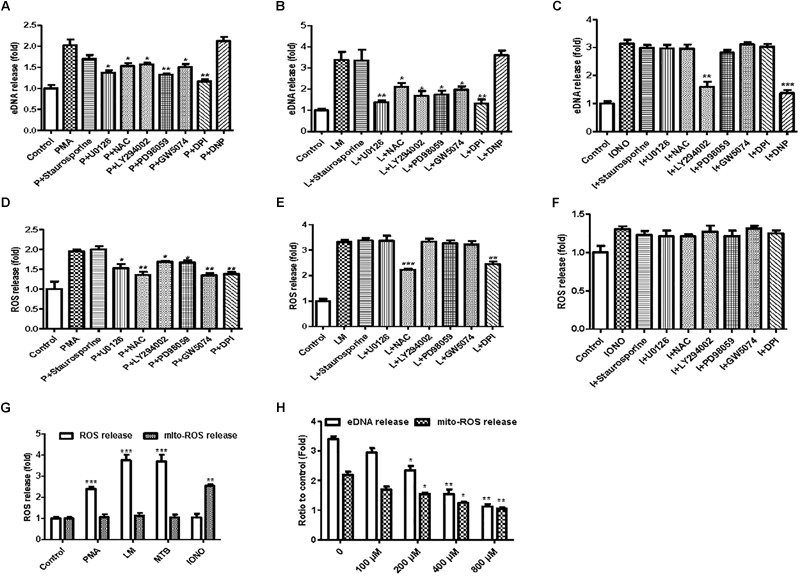
NOX-dependent and NOX-independent MiETs. BV2 cells were pre-treated with staurosporine (10 nM), U0126 (10 μM), PD98059 (20 μM), DPI (50 μM), LY294002 (50 μM), GW5074 (10 nM), or DNP (200 μM) for 1 h, then treated with PMA (2 μM) **(A)**, Listeria (MOI = 50:1) **(B)**, or IONO (1.5 μM) **(C)** for 2 h. Then, eDNA contents were detected. For the quantification of cytosolic ROS release, BV2 cells were pre-treated with similar inhibitors (except DNP) for 1 h and then treated with PMA (2 μM) **(D)**, Listeria (MOI = 50:1) **(E)**, or IONO (1.5 μM) **(F)** for 2 h. Cytosolic ROS and mitochondrial ROS release were detected in PMA-, Listeria- and IONO-treated BV2 cells **(G)**. BV2 cells were pre-treated with a series of concentrations of DNP (100–800 μM) for 1 h and then treated with 1.5 μM ionomycin for 30 min. eDNA and mitochondrial ROS were detected on a plate reader **(H)**. ^∗^*p* < 0.05, ^∗∗^*p* < 0.01, ^∗∗∗^*p* < 0.001, compared with the control cells. The data are representative of triplicate experiments and presented as the mean value ± the SEM.

To investigate the possible mechanisms involved in the formation of MiETs, the NOX inhibitor DPI inhibited the induction of MiETs by Listeria and PMA. In contrast, DPI had little effect on ionomycin-induced MiET release (Figure 6A–C and Supplementary Figures S2A–C). This finding indicated that Listeria- or PMA-induced MiETs were NOX-dependent, whereas ionomycin-induced MiETs were NOX-independent. Next, we used a fluorescent indicator of cytosolic ROS (DHR 123) to detect cytosolic ROS production in microglia. Bacterial infection or PMA treatment caused a large increase in cytosolic ROS production, whereas ionomycin induced little cytosolic ROS production (Figure 6D–F and Supplementary Figures S2D–F). DPI significantly inhibited Listeria- and PMA-induced cytosolic ROS but not affect ionomycin-induced cytosolic ROS production (Figure 6D–F and Supplementary Figures S2D–F). These results demonstrated that NOX-independent MiETs were different from NOX-dependent MiETs.

In order to investigate the pathways involved in MiET formation, we used a variety of inhibitors, including staurosporine (PKC inhibitor), U0126 (MEK inhibitor), PD98059 (ERK inhibitor), DPI (NOX inhibitor), LY294002 (AKT inhibitor), and GW5074 (c-Raf inhibitor). Staurosporine did not significantly reduce the release of cytosolic ROS and the induction of MiETs by Listeria, PMA or ionomycin, suggesting that ROS and MiET release occurred independently of PKC. U0126, LY294002, PD98059, and GW5074 significantly inhibited PMA-induced cytosolic ROS but not bacteria-induced cytosolic ROS production; conversely, these inhibitors prevented MiET release induced by PMA or bacterial treatment. These data confirmed that the Raf-MEK-ERK pathway played a role in MiET formation. PMA- or bacterial-induced MiET production was associated with the production of ROS by NOX, and PMA but not the bacteria were affected by Raf, MEK, ERK and AKT. However, these inhibitors (with the exception of LY294002) did not inhibit ionomycin-induced MiET release (Figure 6C and Supplementary Figure S2C), indicating that NOX-independent MiET induction by ionomycin was mediated by AKT. Thus, PMA-induced cytosolic ROS was inhibited by the ERK inhibitor PD98059 (Figure 6D and Supplementary Figure S2D), ERK functioned upstream of the superoxide formation induced by PMA. In contrast to PMA, bacterial-induced cytosolic ROS was not inhibited by PD98059 (Figure 6E and Supplementary Figure S2E), demonstrating that ERK was downstream of superoxide formation during the bacteria-induced process. Notably, the AKT inhibitor LY294002 did not inhibit cytosolic ROS production by bacteria but had the opposite effect on PMA-induced ROS production.

We also performed a plate reader assay with MitoSOX (a mitochondrial ROS-specific fluorescent dye) to investigate mitochondrial ROS production in MiETs. We found that microglia produced a large amount of mitochondrial ROS following induction by ionomycin but not by Listeria or PMA (Figure 6G and Supplementary Figure S2G). Preincubation with the mitochondrial uncoupler dinitrophenol (DNP) abolished ionomycin-induced mitochondrial ROS production and MiET release in a dose-dependent manner (*p* < 0.05; Figure 6H and Supplementary Figure S2H). However, DNP treatment did not repress bacterial infection- or PMA-induced MiETs (Figure 6A,B and Supplementary Figures S2A,B). This finding indicated that mitochondrial ROS production was necessary for ionomycin-induced NOX-independent MiETs but not for NOX-dependent MiETs.

Immunoblot analysis showed that bacterial infection and PMA treatment led to the strong phosphorylation of ERK in BV2 cells (Supplementary Figures S2I,J). Thus, ERK activation is required for Listeria- or PMA-induced MiET release. Ionomycin caused a very low level of ERK activation (Supplementary Figure S2K). Pretreatment with staurosporine did not significantly reduce bacteria- or PMA-induced ERK phosphorylation in microglia (Supplementary Figures S2I,J). DPI significantly blocked bacteria-induced ERK phosphorylation but could not prevent PMA-induced ERK phosphorylation. This result verified that ERK activation occurred downstream of superoxide generation following bacterial infection but not PMA treatment. U0126, LY294002, PD98059, and GW5074 significantly inhibited the phosphorylation of ERK by PMA treatment and bacterial infection (Supplementary Figures S2I,J). Overall, these data demonstrated that bacteria and PMA induced NOX-dependent MiETs, while ionomycin induced NOX-independent MiETs. NOX-dependent MiETs required both ROS generation and ERK activation. ERK activation was mediated by raf-MEK-ERK activation, whereas mitochondrial ROS and AKT participated in NOX-independent MiETs.

### The Effect of Interferon-γ on MiET Production *in vitro*

**FIGURE 7:**
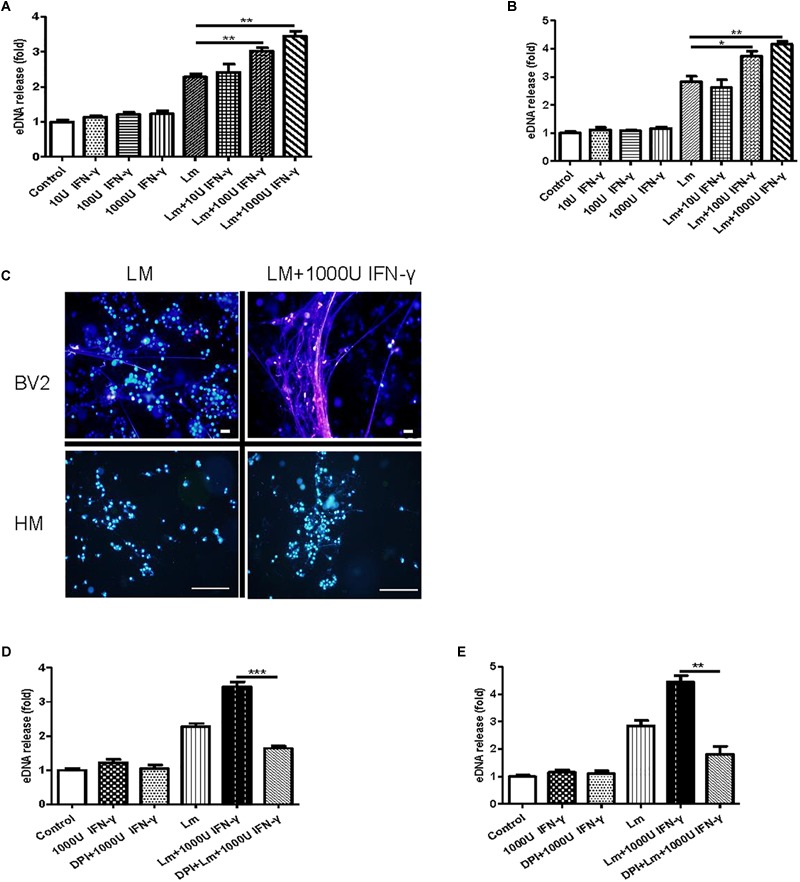
The effect of interferon-γ on MiET production *in vitro*. Mouse or human microglia were incubated in 24-well flat bottom plates. BV2 cells were treated with diluted concentrations of IFN-γ, IFN-γ and Listeria; then, eDNA release was detected **(A)**. Human microglia were treated the same as described for BV2 cells **(B)**. eDNA staining was detected **(C)**. Scale bars = 50 μm. BV2 cells were pre-treated with DPI (50 μM) for 1 h and then treated with IFN-γ (1000 U), IFN-γ (1000 U) and Listeria for 2 h. A plate reader was used to quantify eDNA release **(D)**. Human microglia were conducted as described above for BV2 cells **(E)**. ^∗^*p* < 0.05, ^∗∗^*p* < 0.01, ^∗∗∗^*p* < 0.001. The data are representative of triplicate experiments and presented as the mean value ± the SEM.

A recent report demonstrated that bacteria exploited interferon-γ to excite human macrophage ET formation (Douda et al., 2015). To investigate whether IFN-γ played a role in stimulating MiET formation, we detected the effect of Listeria and PMA on interferon-γ levels in mouse and human microglia *in vitro*. The results showed that the IFN-γ contents of supernatants from mouse and human microglia treated with the two agents were all below the limit of detection, suggesting that IFN-γ was not released to a significant extent (date not shown). Notably, treatment with interferon-γ induced more eDNA release from both mouse and human microglia *in vitro* compared with the control, although this increase did not reach statistical significance (*p* > 0.05, Figure 7A,B). Intriguingly, interferon-γ treatment significantly improved MiET formation in Listeria-infected microglia in a concentration-dependent manner (10–1000 U/ml) (Figure 7A,B). These results were confirmed by Hoechst 33342 staining (Figure 7C). Furthermore, pretreatment with the NOX inhibitor DPI abolished MiET induction by interferon-γ or the combination of interferon-γ and bacteria (Figure 7D,E). This finding suggested that the induction of MiETs by interferon-γ was mediated by NOX. We hypothesized that interferon-γ might promote ROS production, thereby leading to MiET formation. A recent report showed that the bacterial component LPS could enhance ROS production induced by interferon-γ in microglia (Wong and Jacobs, 2013), which was in agreement with our results.

### *In vivo* and *in vitro* MiET Formation and Bacterial Killing in a Mouse Meningitis Model

**FIGURE 8:**
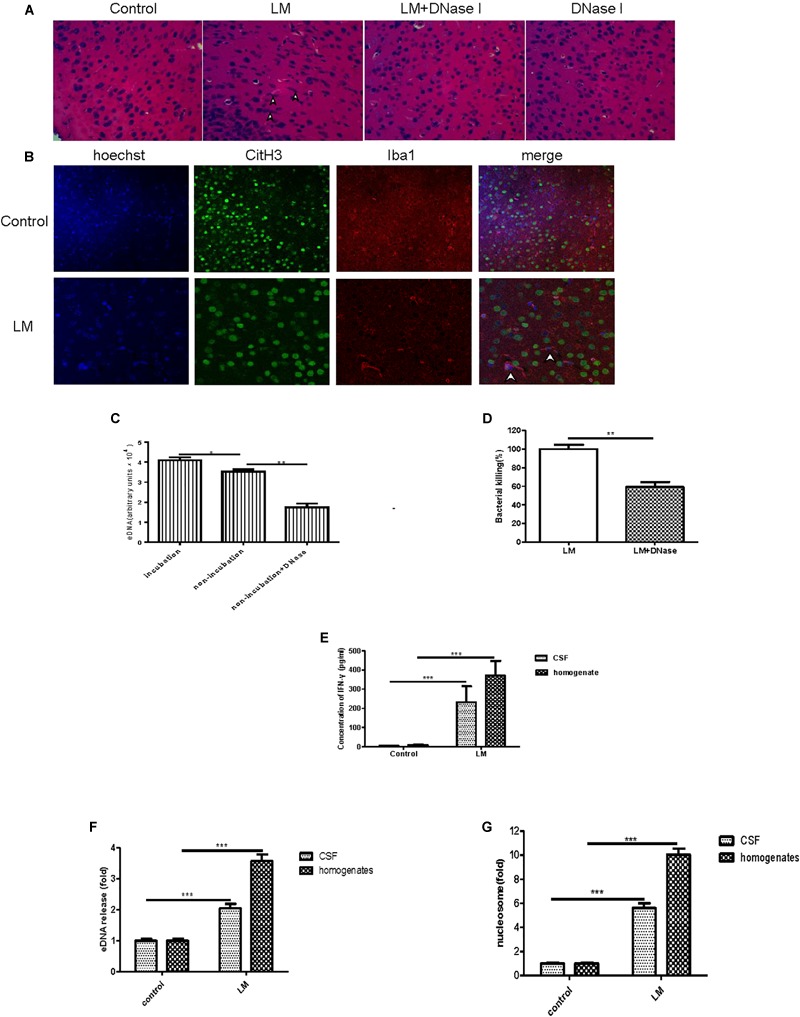
*In vivo* and *in vitro* MiET formation and bacterial killing in a mouse meningitis model. The brain parenchyma of mice was injected with Listeria (1 × 10^5^) or DNase I; PBS injection was used as a control. After 24 h, the mice were euthanized to obtain their brains for paraffin sections. H&E staining of brain tissue slices was observed by microscope with a 40 × objective lens **(A)**. Colocalization of histone (H3 antibody), microglia (Iba-1 antibody), and eDNA (Hoechst 33342) was observed by confocal microscopy with 20 or 40 × objective lens **(B)**. eDNA contents were detected after the addition of DNase I (40 U/ml) to the CSF samples **(C)**. The Listeria meningitis mouse model was established for 24 h with or without DNaseI (5 μg), then, the bacteria killing rate was calculated **(D)** and the level of IFN-γ in both the CSF and homogenates was detected **(E)**. The level of eDNA release in CSF and homogenates **(F)** from Listeria -infected mice were detected with a plate reader following Sytox green staining. The nucleosome contents of the CSF and homogenates **(G)** were detected with an ELISA kit. ^∗^*p* < 0.05, ^∗^*^∗^p* < 0.01, ^∗∗∗^*p* < 0.001. The data are representative of triplicate experiments and presented as the mean value ± the SEM.

To seek *in vivo* evidence of MiET formation and Listeria killing by MiETs, we established Listeria meningitis mouse models. All infected animals developed clinical signs of bacterial meningitis until 24 h after infection including aggressive activity and decreased food intake. But no mice died before the time of sacrifice (1 day after infection). H&E staining of brain tissue slices revealed the existence of eDNA in the brain cerebral cortex, and there were much more inflammatory infiltrates (leukocyte infiltration, neurophagy and encephalomalacia foci formation), and less ET exhibition in murine brain of DNase I-treated infected group, while historical inflammatory changes were not so heavy but MiETs were easily observed in infected mice (Figure 8A). The results indicated that MiET formation had the potential to protect the bacteria infection in the CNS. Immunofluorescence staining indicated that there was colocalization between histone (H3 antibody), microglia (Iba-1 antibody), and eDNA, which confirmed MiET formation in brain tissue slices in Listeria-infected mice (Figure 8B). To quantify bacterial-induced MiETs *in vivo*, we incubated non-centrifuged CSF on chamber slides to allow the ETs to adhere. The PADs inhibitor Cl-amidine was used to block *in vitro M*iET formation during incubation. Representative fluorescence microscopy images showed the presence of some MiET fibres and colocalization of microglia and eDNA in *in vivo*-generated (Cl-amidine -treated CSF) ETs (Supplementary Figure S3A). Incubation of (non-centrifuged) CSF without Cl-amidine (*in vitro* ETs) resulted in the continued formation of additional MiETs (Supplementary Figure S3B). These results corroborated that MiET formation occurred *in vivo* in the infection setting.

Next, we assessed the killing of bacteria *in vivo* by MiETs. First, our *in vitro* experiment confirmed that DNase I did not possess antibacterial activities (data not shown). It was found that exogenous DNase I treatment significantly decreased eDNA release in non-incubated non-centrifuged CSF from both Listeria-infected mice (Figure 8C). Furthermore, DNase I treatment led to an approximately 62% decrease in Listeria killing of mouse brain homogenates 24 h after infection (Figure 8D). Brain homogenates and the CSF of control mice injected with saline did not contain bacteria, and their low Listeria CFUs were detected in their CSF cultures. It’s worth mentioning that in the group of Listeria-infected mice with DNase I treatment, the median survival time is 2.6 days and significantly shorter than the infected with Listeria without DNase I treatment group (4 days) (*p* < 0.05) or treated with DNase I only group (>5 days) (*p* < 0.001) (Supplementary Figure S3C). Together, all these results suggested that MiET formation protected against Listeria infection of the CNS.

The levels of IFN-γ in brain homogenates and CSF were significantly higher in the Listeria-infected mice compared to the non-injected controls (Figure 8E). The levels of both eDNA and nucleosomes in brain CSF (Figure 8F) and homogenates (Figure 8G) were significantly higher in the Listeria-infected mice compared to the healthy controls. Finally, there was a positive relationship between the level of eDNA and nucleosomes (*r* = 0.94), IFN-γ and eDNA (*r* = 0.92), IFN-γ and nucleosome (*r* = 0.91) in the brains of the Listeria meningitis model mice.

### Detection of MiETs in CSF From Listeria Meningitis Patients

To assess MiET formation *in vivo* and its relative index, we collected CSF samples from 9 Listeria meningitis patients [including 7 adults and 2 children (mean age, 30 months)], and 6 adult healthy volunteers. To rule out the effect of antibacterial chemotherapy on the testing result, these CSF samples were collected prior to treatment. To confirm the existence of MiETs in human BM, we incubated non-centrifuged patient CSF in chamber slides. Both *in vivo*-generated (Cl-amidine treated CSF) (Supplementary Figure S4A) and *in vitro-*generated (CSF without Cl-amidine treatment) ETs were found in CSF samples from Listeria patients (Supplementary Figure S4B). Colocalization between microglia and eDNA was detected in these ETs (Supplementary Figures S4A,B). These findings demonstrated that MiETs were formed in intracranial human BM.

Next, the levels of IFN-γ in the CSF of Listeria patients were measured. Our results showed that all 9 BM patients had markedly elevated levels of IFN-γ in the CSF compared to the healthy controls (*p* < 0.01, Supplementary Figure S4C). The levels of both eDNA and nucleosomes were significantly higher in the 9 BM patients compared to the healthy controls (Supplementary Figures S4D,E). There was a significant relationship between the nucleosome and eDNA concentrations (*r* = 0.93), IFN-γ and eDNA concentrations (*r* = 0.91), IFN-γ and nucleosome concentrations (*r* = 0.91) in the CSF from the Listeria patients. Overall, Listeria were found to generate high levels of MiETs *in vivo* in BM patients, which validates the MiET-stimulatory capacity of BM *in vitro* and *in vivo* model mice.

## Discussion

Meningitis due to Listeria is a severe invasive infectious disease with a high fatality rate in persons at risk (Lisi et al., 2014). A previous study showed that microglia are relevant target cells for Listeria in the CNS; however, microglia are significant in cerebral listeriosis to avoid pathogen dissemination in the CNS and preserve brain homeostasis (Virna et al., 2006). Moreover, CD8^+^ T cells are involved in the primary response and are especially important for protective immunity to Listeria infections, and intracellular bacteria-infected cells could be lysed by CD8^+^ T cells (Lim et al., 2017). Our results expounded that Listeria could directly stimulate microglia to produce MiETs and be entrapped and killed by MiETs without transmission from cell to cell, a phenomenon was confirmed by our results (Figure 1D). In addition, we speculated that Listeria spread between microglia similar to macrophages, subsequently exposing the bacteria to the extracellular space under the work of CD8^+^ T cells. The released bacteria induce microglia to produce vesicle-releasing MiETs, and then the bacteria are entrapped and killed by MiETs. Our results had demonstrated the phenomenon *in vitro* (Figure 2), and we will complete further experiments *in vivo* related to it in the future.

As important phagocytes in the CNS, microglia have been shown to possess antibacterial activity and some innate antibacterial mechanisms such as autophagy, ROS production, and inflammasome production (van Meijgaarden et al., 2015). In this study, we demonstrated that Listeria-, PMA- and ionomycin-induced MiET formation is a common phenomenon in three types of microglia BV2 cells, primary mouse microglia, and human microglia. Furthermore, MiET could entrap and kill extracellular Listeria.

Moreover, we showed that eDNA, MMP9, MMP12, citrullinated histone H3 and PAD2 were the components of MiETs; microglia have been reported to highly produce MMP9, MMP12 and PAD2 (Maeda and Sobel, 1996; Asaga et al., 2002; Chakraborty et al., 2010), whereas MMP9 and MMP12 were not reported in NETs (Brinkmann et al., 2004). DNase I dissociated ETs, and Cl-amidine decreased histone hypercitrullination and abrogated the formation of Listeria- or PMA-induced MiETs. This result suggests that histone hypercitrullination mediated MiET formation, a finding that was consistent with that reported for NET formation (Brinkmann et al., 2004). Because the bactericidal activity of MiET is of more interest than MiET formation itself, our results showed that MiETs could kill Listeria *in vitro*. DNase I, Cl-amidine and the addition of anti-histone significantly decreased the killing of extracellular Listeria. Bactericidal properties have been demonstrated for histones in mammals (Brinkmann et al., 2004). However, we speculated that other bactericidal elements in addition to histones might exist in MiETs. It was previously reported that vesicles play a role in the interaction between cells and the host pathogen (Bianco et al., 2005). In this study, we verified that microglia produce vesicles following Listeria infection, and the vesicles release their contents to form MiETs. Notably, the DNA-containing vesicles are isolated from the nucleus, appearing outside of the cell and releasing DNA into the extracellular space. This discovery is similar to the previously reported formation of NETs (Garzetti et al., 2014). In addition, MiETs and vesicles have similar compositions, and the main components of MiETs are different from those of NETs.

Being as a type of macrophage, microglia could be polarized to different forms under various external stimulators. M1 markers include CD86, CCR7 and TNF-α, whereas M2 markers include CD206, Arg-1 and IL-10. Moreover, Cheng et al. indicated that M1-macrophage vesicles induce a stronger cytotoxic T-cell response and promoted the production of proinflammatory cytokines (Ito et al., 2007). In this study, our results showed that Listeria-induced MiETs cause M1-like polarization in microglia. This discovery confirmed that MiETs are pro-inflammatory during the course of Listeria infection.

Our *in vivo* study investigated MiET formation under physiological conditions. The results confirmed MiET formation in Listeria-infected mice, and exogenous DNase I treatment significantly decreased MiET production *in situ* in Listeria-infected mouse brains or CSF from Listeria-infected mice, affirming the physiological association of MiET formation with the meningitis model. To date, no special inhibitors of ETosis are available. The exogenous DNase used here was previously utilized to destroy NETs (Cheng et al., 2017). Our DNaseI treatment confirmed that MiETs possess bactericidal activity against bacteria *in vivo*.

To date, two main types of ETs have been reported: NOX-dependent and NOX-independent ETs. The NOX-dependent pathway is mainly related to cytosolic ROS, whereas the NOX-independent pathway uses mitochondrial ROS (Yipp et al., 2012). Our results indicated that bacteria- or PMA-induced MiETs are NOX dependent, whereas ionomycin-induced MiETs are NOX independent. Importantly, activated ROS production via NOX has been reported in microglia (Liu et al., 2010). Our results also demonstrated that mitochondrial ROS production is necessary for ionomycin-induced NOX-independent MiET production but not for NOX-dependent MiET production. Recent reports have demonstrated an association between ERK and NOX-dependent NETosis in neutrophils (Hakkim et al., 2011). By contrast, ERK is activated at low levels in NOX-independent NETosis (Yipp et al., 2012). To explore whether the classical Raf-MEK-ERK pathway is involved in MiET formation, our results showed that specific c-Raf, MEK, and ERK inhibitors affect NOX-dependent MiET formation. Additionally, western blotting analysis confirmed that ERK participates in the NOX-dependent pathway. The AKT inhibitor LYS294002 also inhibits MiET formation and the phosphorylation of ERK by PMA or bacteria, indicating that AKT functions upstream of ERK in the MiET formation pathway. By contrast, the PKC inhibitor had no effect on PMA- or bacteria-induced MiET formation, ROS generation, or ERK phosphorylation. Thus, our results proposed that ERK is downstream of NOX-dependent ROS generation, and AKT is localized between ERK and ROS in bacteria-induced MiET formation. Moreover, LY294002 inhibited the phosphorylation of ERK induced by PMA, and PMA-induced cytosolic ROS was inhibited by PD98059, indicating that ERK functions upstream of ROS generation and downstream of AKT in PMA-induced MiET formation.

IFN-γ is thought to be involved in ETs produced by pathogenic microorganism-infected neutrophils or macrophages. In our *in vitro* study, interferon-γ treatment significantly improved Listeria-induced MiET production, although treatment with only interferon-γ induced more (but not significant) eDNA release. The DPI inhibition assay indicated that interferon-γ-induced MiETs are NOX mediated. IFN-γ levels were significantly increased in Listeria-infected mice; these data were in agreement with those of previous reports (Virna et al., 2006). Notably, the IFN-γ levels in the CSF of all Listeria meningitis patients were elevated prior to treatment. This result was in agreement with reports that there were high IFN-γ levels in the CSF of Listeria meningitis patients (Glimaker et al., 1994).

Additionally, elevated eDNA and nucleosome levels were detected in the CSF of Listeria-infected mice and 9 clinical patients. Savchenko et al. (2014) showed that nucleosomes could be released from dying cells or activated inflammatory cells in the form of ETs. To the best of our knowledge, this is first report concerning nucleosomes in CSF from BM subjects. We found that there was a positive relationship between the levels of eDNA and nucleosomes in the CSF of Listeria meningitis patients prior to antimicrobial treatment, suggesting that eDNA and nucleosomes may be used as diagnostic markers for clinical Listeria meningitis. However, whether they are suited to other BM types requires further corroboration. Thus, we summarize the schematic graphs (Supplementary Figure S5) and four highlights in this study. First, Listeria could transmit between microglia, and CD8^+^ T cells could break these infected cells; subsequently, ambient microglia release ETs captured and kill the bacteria. Second, MiETs originate from Listeria-induced microglial vesicles. Third, the main components of MiETs are eDNA, MMP9, MMP12, citrullinated histone H3 and PAD2, which are not identical to the components of NETs. Fourth, eDNA and nucleosomes may be potential diagnostic markers for Listeria meningitis.

Recently, more attention has been given to the role of microglia in CNS disorders. The present study demonstrated that microglia could release ETs to protect against Listeria infection of the CNS. Although the entrapment and killing of Listeria by ETs is favorable, recent reports have demonstrated that ET formation can lead to vascular disorders (Kaplan and Radic, 2012) or thrombus formation. Thus, a deeper exploration of MiETs is warranted based on the findings of this study.

## Ethics Statement

Mice were maintained under standard conditions according to our institutional guidelines. The experiments were approved by the Jilin University Animal Care and Use Committee (No. IZ-2009-008). Cerebrospinal fluid (CSF) samples (*n* = 9) were collected from patients reporting to the First Hospital of Jilin University and Changchun Infectious Disease Hospital. Bacterial meningitis (BM) was diagnosed through bacterial culture, bacterial smears. Nine patients were diagnosed with Listeria meningitis; additionally, six healthy control volunteers were included. The procedures were in agreement with the regulations and ethical standards of the two hospitals for the collection of specimens and basic clinical information, written informed consent was obtained from all patients who participated in this study.

## Author Contributions

LY, CW, and XS participated design of this study, and performed the statistical analysis. LY carried out the study and collected important background information. CW and XS drafted the manuscript. XS, CW, XT, and YW carried out the concepts, design, definition of intellectual content, literature search and data analysis, and prepared the manuscript. WC, XueyanW, YA, SL, HX, YL, XuefeiW, WL, and ZC provided assistance for data acquisition, data analysis, and statistical analysis. ML performed manuscript review. All authors read and approved the final content of the manuscript and provided consent in written form.

## Conflict of Interest Statement

The authors declare that the research was conducted in the absence of any commercial or financial relationships that could be construed as a potential conflict of interest.
