# Supplementary material for: The TRAPs From Microglial Vesicles Protect Against Listeria Infection in the CNS
Source: Front Cell Neurosci. 2019 May 7;13:199. doi: 10.3389/fncel.2019.00199 (PMC6516055; doi:10.3389/fncel.2019.00199)
Supplement: Supplementary file 1 [file Table_1.DOCX]

Table S1. Reagent information

| Reagent | Concentration | Company |
| --- | --- | --- |
| PMA | 2 µM | MCE |
| Ionomycin (IONO) | 1.5 µM | MCE |
| Staurosporine | 10 nM | MCE |
| U0126 | 10 µM | MCE |
| PD98059 | 20 µM | MCE |
| DPI | 50 µM | MCE |
| LY294002 | 50 µM | MCE |
| GW5074 | 10 nM | MCE |
| FITC | 5 μM | MCE |
| Hoechst 33342 | 10 μM | MCE |
| Cl-amidine | 100 µM | MCE |
| Dinitrophenol (DNP) | 200 µM | TCI AMERICA |
| β-hydroxybutyrate (BHBA) | 4 mM | TCI AMERICA |
| DNase I | 40 U/ml | Invitrogen |
| SYTOX Orange | 5 μM | Invitrogen |
| Dihydrorhodamine 123 (DHR 123) | 5 μM | Sigma-Aldrich |
| MitoSOX Red | 5 μM | Molecular Probes |

**Antibody**

Rabbit anti-mouse CD86-PE, Rabbit anti-mouse CD206-FITC, Rabbit anti-mouse CD9-AF647, Mouse anti-Human CD8a-Cy7 were purchased from BD Biosciences.

Phalloidin-iFluor 594, CD9 antibody, CD81 antibody, MMP9 antibody, MMP12 antibody, IFN-γ antibody, Iba1 antibody, CCR7 antibody, Arg-1 antibody, ERK antibody, p-ERK antibody, PAD2 antibody, Cit-histone H3 antibody, β-actin antibody, histone H2A antibody and IL-1β antibody were purchased from Abcam.

Table S2. Clinical presentations of the nine cases

| Case No. | Age | Gender | Fever | Headache | Meningeal signs | Nausea/vomiting | Medical History |
| --- | --- | --- | --- | --- | --- | --- | --- |
| 1 | 63 | Male | Pos. | Pos. | Pos. | Pos. | None |
| 2 | 68 | Male | Pos. | Pos. | Neg. | Pos. | Diabetes |
| 3 | 35 | Male | Pos. | Pos. | Pos. | Pos. | None |
| 4 | 47 | Male | Pos. | Neg. | Pos. | Neg. | None |
| 5 | 40 | Female | Pos. | Pos. | Pos. | Pos. | SLE |
| 6 | 18 | Female | Pos. | Pos. | Pos. | Pos. | None |
| 7 | 59 | Male | Pos. | Pos. | Pos. | Neg. | None |
| 8 | 63 | Female | Pos. | Neg. | Pos. | Pos. | AOSD |
| 9 | 65 | Male | Pos. | Pos. | Neg. | Pos. | Diabetes |
| Pos.: positive, Neg.: negative, SLE: systemic lupus erythematosus, AOSD: adult-onset Still's disease | | | | | | | |
