# Supplementary figures and images for: The TRAPs From Microglial Vesicles Protect Against Listeria Infection in the CNS
Source: Front Cell Neurosci. 2019 May 7;13:199. doi: 10.3389/fncel.2019.00199 (PMC6516055; doi:10.3389/fncel.2019.00199)

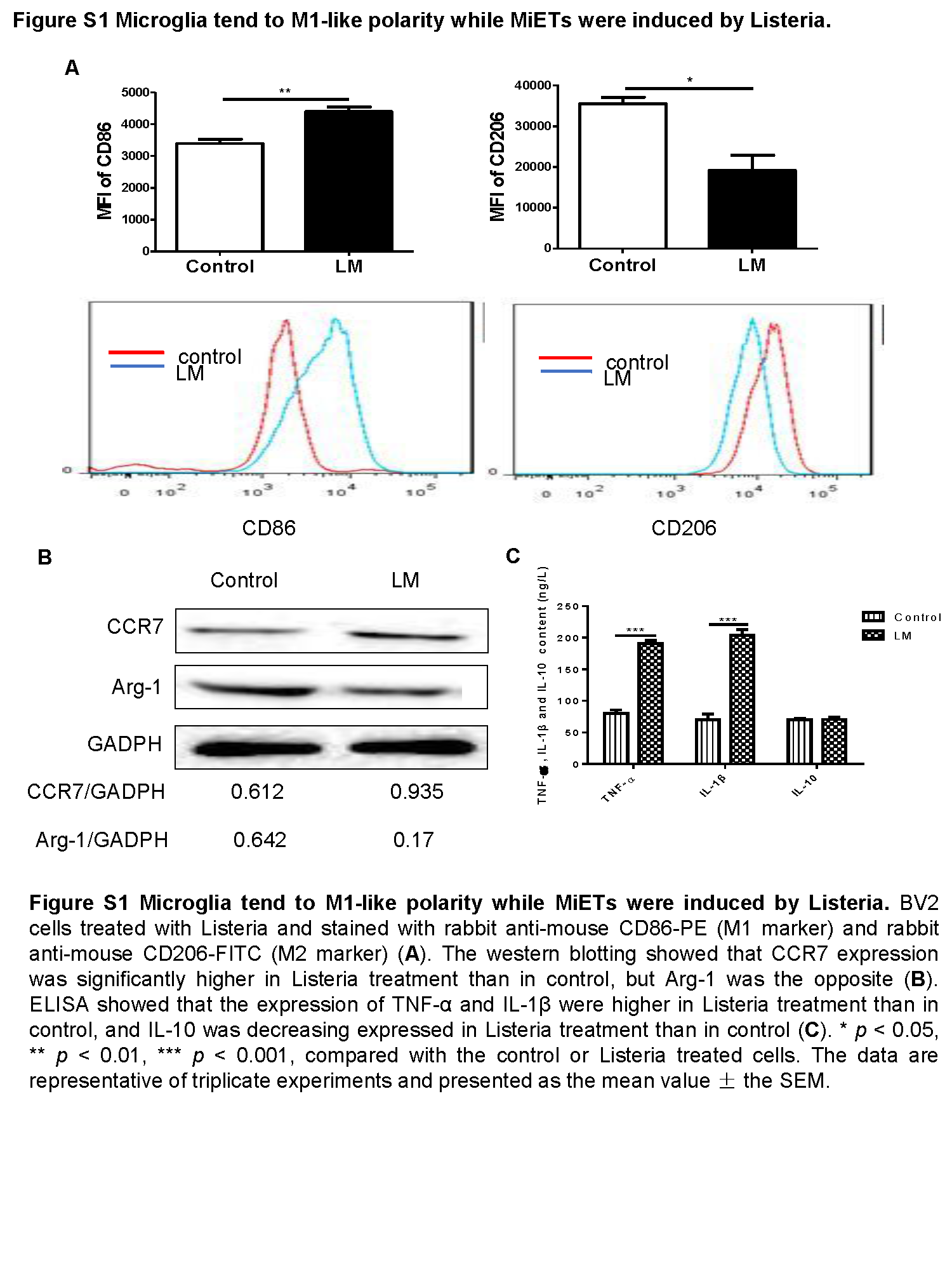

Supplement: Supplementary file 2 [file Image_1.TIFF]

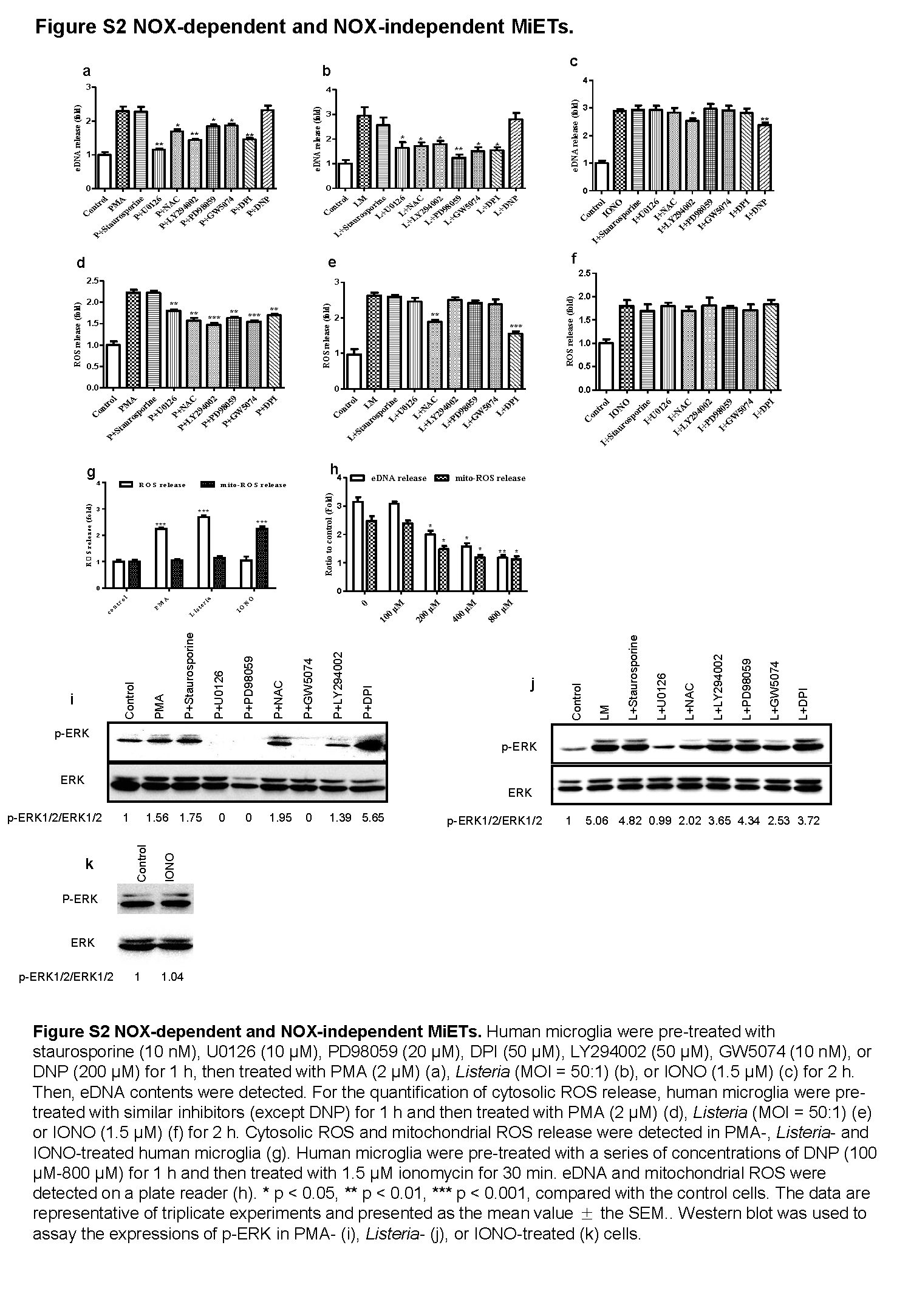

Supplement: Supplementary file 3 [file Image_2.TIFF]

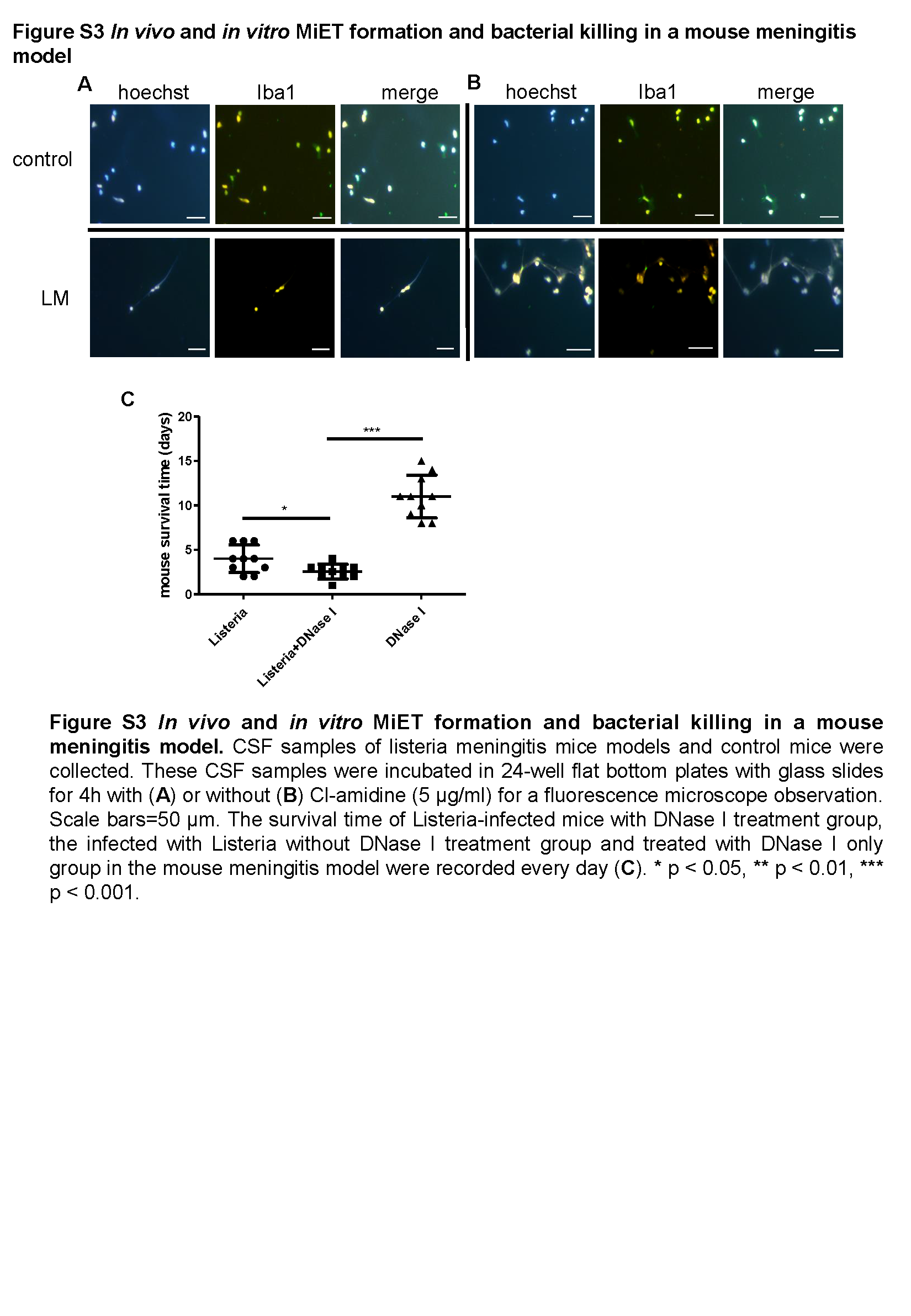

Supplement: Supplementary file 4 [file Image_3.TIFF]

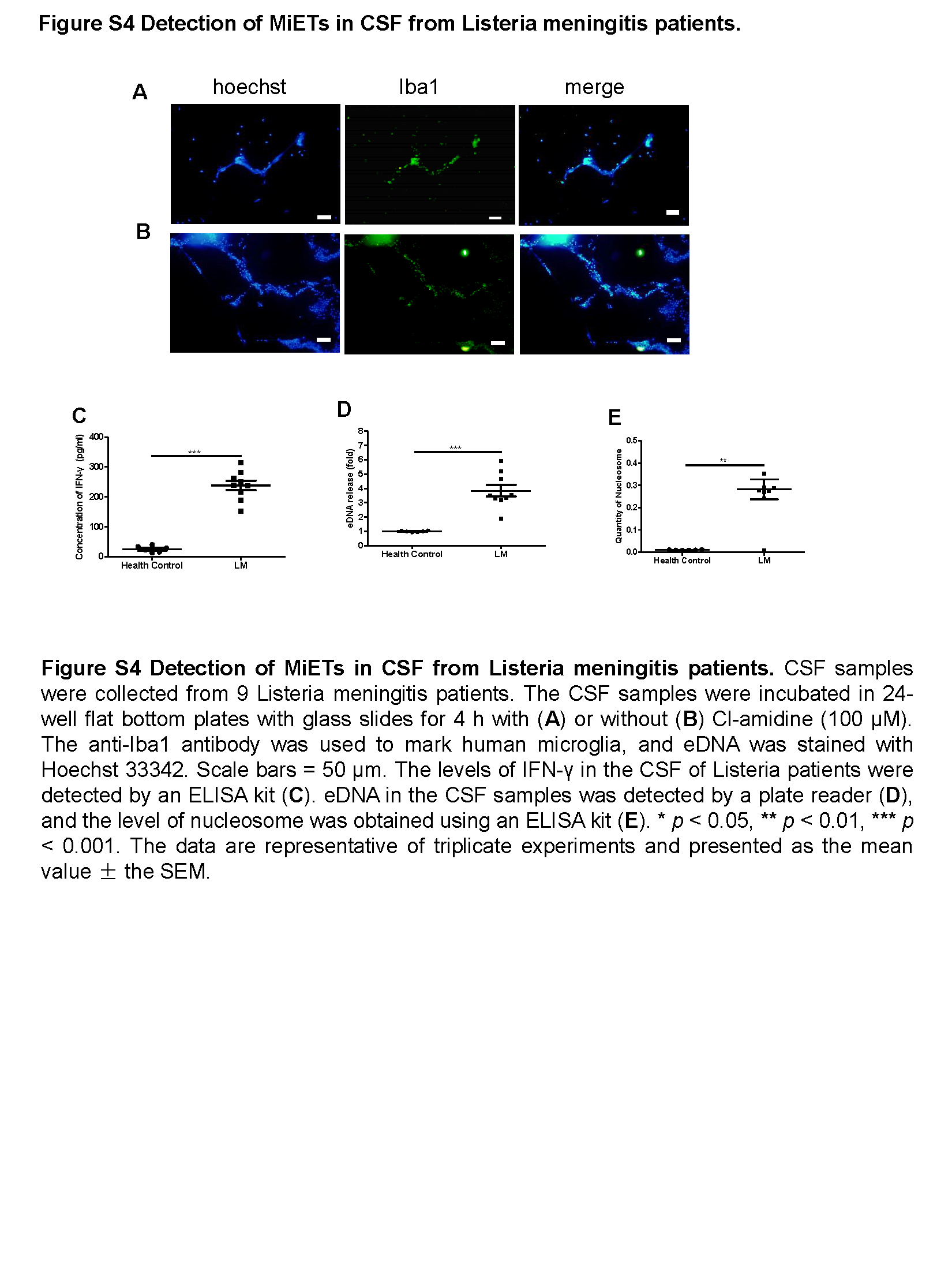

Supplement: Supplementary file 5 [file Image_4.TIFF]

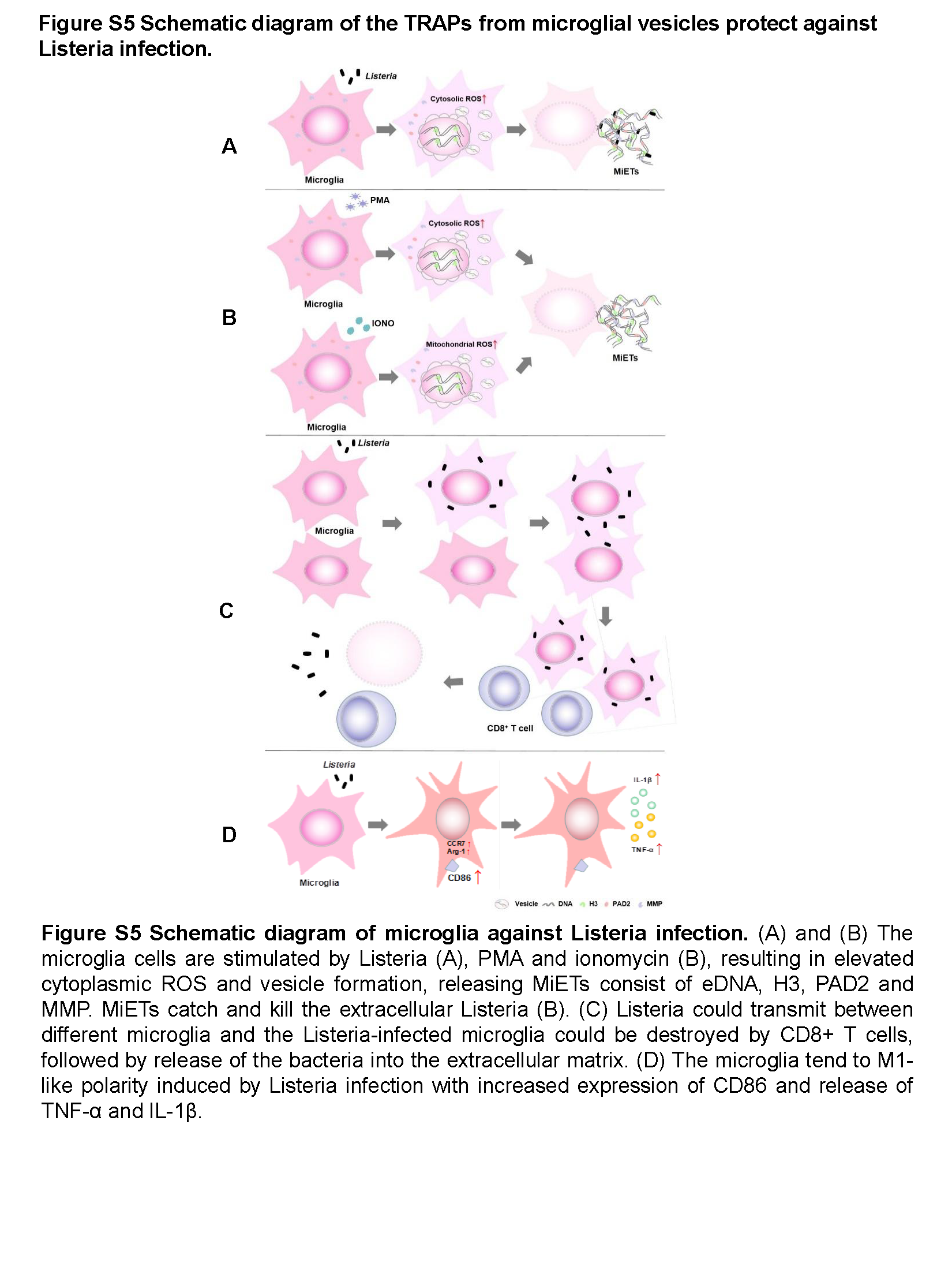

Supplement: Supplementary file 6 [file Image_5.TIFF]
